# Spontaneous defervescence and its prediction in the acute phase of Kawasaki disease

**DOI:** 10.3389/fped.2022.943203

**Published:** 2022-08-04

**Authors:** Eun Jung Shin, Jeong Jin Yu, Hyewon Shin, Hyuck Jin Kwon, Jin Ho Kim, Mi Jin Kim, Seulgi Cha, Jae Suk Baek

**Affiliations:** ^1^Department of Pediatrics, Inha University College of Medicine, Incheon, South Korea; ^2^Department of Pediatrics, Asan Medical Center, University of Ulsan College of Medicine, Seoul, South Korea

**Keywords:** aspirin, children, defervescence, fever, Kawasaki disease

## Abstract

**Background:**

In Kawasaki disease (KD), fever occasionally resolves spontaneously before 10 days from the onset, right after diagnosing. However, there is not enough evidence of intravenous immunoglobulin (IVIG) treatment in this case. The aim of this study was to investigate the relationship between spontaneous defervescence and coronary artery aneurysm and to develop a scoring model for its prediction in acute KD.

**Methods:**

All patients admitted for acute KD in Asan Medical Center were considered for inclusion. Acute management involved the administration of 2 g/kg of IVIG and 5 mg/kg/day of aspirin. The patient whose temperature was <37.5°C for more than 48 h from the diagnosis was discharged under the judgment of spontaneous defervescence, without IVIG administration.

**Results:**

The incidence of coronary artery aneurysm was 5.7% in 94 defervesced patients and 4.6% in the 1,277 patients treated with IVIG in the subacute phase (*P* = 0.593), and 2.5 and 2.2% in respective patient groups in the convalescent phase (*P* = 0.924). A scoring model which predicted spontaneous defervescence under the combination of C-reactive protein ≤10mg/dL and ≥2 conditions of no rash, neutrophil ≤65%, and/or alanine aminotransferase ≤80 IU/L, was developed and showed 80.7% sensitivity, 68.8% specificity, 15.8% positive predictive value, and a 97.8% negative predictive value.

**Conclusion:**

The incidence of coronary artery aneurysm in patients with the defervesced KD was not different from the IVIG treated patients. In the cases suitable for the predictive model, patients can wait for the spontaneous defervescence under intensive observation by medical professionals.

## Introduction

Kawasaki disease is an acute febrile vasculitis of unidentified etiology. Although it is self-limiting, coronary artery aneurysm (CAA) has been reported in up to 25% of untreated cases and therefore timely treatment is essential (1). It is recommended that intravenous immunoglobulin (IVIG), an effective initial treatment, be administered within 10 days after the onset of fever (1). According to the treatment guidelines, IVIG administration should be started as soon as possible, especially if fever is present (1–3). Therefore, timely recognition of Kawasaki disease is so important in the management during acute phase. However, fever occasionally resolves spontaneously 10 days after the onset in some patients. According to the guidelines (1–3), IVIG does not need to be administered in patients with spontaneous defervescence under additional conditions—normalization of laboratory values and normal echocardiographic results (1, 3) or low severity of the disease (2). However, there is not enough evidence for these recommendations, and reports of patients’ outcome with spontaneously defervesced illness is limited.

So far, there are two studies on spontaneous defervescence in acute Kawasaki disease (4, 5). In these studies, coronary complications in patients with spontaneously defervesced Kawasaki disease ranged from 9.9 to 18.9% (4, 5). In addition, there is a case report of a baby suspected of incomplete Kawasaki disease who defervesced without IVIG treatment but had a giant coronary aneurysm (6).

This study aimed to investigate the relationship between spontaneous defervescence and coronary complications and develop a scoring model for the prediction of fever subsidence in acute Kawasaki disease.

## Materials and methods

### Subjects

All the patients admitted to the Asan Medical Center for the treatment of acute Kawasaki disease between January 2006 and December 2019, were considered for inclusion in the study. Patients diagnosed after 10 days of fever or who were not followed up after the acute phase were excluded.

A diagnosis of Kawasaki disease was made in accordance with the 2004 American Heart Association guidelines (7). Incomplete presentation was defined as a case with unexplained fever for ≥5 days associated with 2 or 3 of the principal clinical features (Table 1). The acute care for Kawasaki disease in our center is 2 g/kg of IVIG and 5 mg/kg/day of low-dose aspirin. Medium- or higher-dose of aspirin (≥30 mg/kg/day) is not administered regardless of the phase of illness. After diagnosis, the patients received IVIG treatment if the body temperature was ≥37.5°C. Corticosteroids were administered only in patients who did not respond to IVIG treatment. The definition of spontaneous defervescence was a body temperature lower than 37.5°C for more than 48 h after arriving at the hospital. Patients with spontaneous defervescence were discharged without the administration of IVIG. All the patients were recommended to take body temperature measurements at least three times a day after discharge and re-visit the hospital if the temperature was ≥38.0°C or if a mild fever (37.5–38.0°C) occurred ≥2 times a day.

**TABLE 1:** Characteristics of the subjects.

Characteristics	Defervesced KD (*n* = 94)	Treated KD (*n* = 1277)	*P*-value
Age, years	2.48 ± 2.05	2.64 ± 2.0	0.477
Male	58 (61.1)	746 (58.4)	0.533
Family history	0 (0.0)	10 (0.8)	0.389
Recurrence	0 (0.0)	53 (4.2)	0.044
Body weight, kg	13.7 ± 9.8	13.6 ± 5.8	0.841
Height, cm	87.3 ± 18.8	90.5 ± 16.7	0.071
***Principal clinical features***			
Conjunctivitis	88 (92.6)	1,222 (95.7)	0.346
Red lips/oral mucosa	82 (86.3)	1,170 (91.6)	0.145
Rash	69 (72.6)	1,106 (86.6)	<0.001
Cervical lymphadenopathy	54 (56.8)	832 (65.2)	0.132
Changes in extremities	76 (80.0)	1,119 (87.6)	0.058
Complete presentation	72 (75.8)	1,116 (87.4)	0.003
BCG site reaction	46 (51.7)	569 (49.3)	0.516
Days of illness at diagnosis	5.3 ± 1.7	5.5 ± 1.4	0.185
***Laboratory findings***			
WBC, × 10^3^/μL	11.6 ± 4.3	13.6 ± 4.8	<0.001
Neutrophil,%	46.8 ± 15.8	62.7 ± 16.0	<0.001
Hemoglobin, g/dL	11.7 ± 1.1	11.3 ± 1.1	0.003
Hematocrit	34.6 ± 3.2	34.2 ± 3.1	0.217
Platelet, × 10^3^/μL	337.5 ± 122.1	347.2 ± 107.6	0.411
Protein, g/dL	6.7 ± 0.6	6.7 ± 0.7	0.865
Albumin, g/dL	3.6 ± 0.4	3.4 ± 0.5	<0.001
AST, IU/L	38.7 ± 28.9	77.5 ± 131.2	0.004
ALT, IU/L	33.8 ± 60.8	94.1 ± 137.6	<0.001
Total bilirubin, mg/dL	0.41 ± 0.41	0.68 ± 0.81	0.001
Na^+^, mmol/L	137.7 ± 2.4	136.3 ± 2.8	<0.001
C-reactive protein, mg/dL	3.30 ± 3.06	8.47 ± 6.32	<0.001
Pyuria	11 (12.6)	329 (31.0)	<0.001
***Coronary artery status before initial IVIG***			
LMCA, mm	2.39 ± 0.58	2.34 ± 0.48	0.375
Z score	0.50 ± 1.15	0.40 ± 1.01	0.388
LAD, mm	1.82 ± 0.41	1.91 ± 0.46	0.075
Z score	0.15 ± 1.05	0.40 ± 1.16	0.062
RCA, mm	1.83 ± 0.41	1.99 ± 0.49	0.004
Z score	–0.18 ± 1.0	0.22 ± 1.05	0.001
CAA	7 (8.0)	32 (4.3)	0.124

Data are presented as mean ± standard deviation, or number (%).

KD, Kawasaki disease; BCG, Bacillus Calmette–Guérin vaccine; WBC, white blood cell; AST, aspartate aminotransferase; ALT, alanine aminotransferase; LMCA, left main coronary artery; LAD, left anterior descending; RCA, right coronary artery; CAA, coronary artery aneurysm.

### Study design

Subjects with the initial confirmation of spontaneous defervescence were classified into the defervesced Kawasaki disease group, and the other subjects who received the initial IVIG treatment were included in the treated group. Among the defervesced Kawasaki disease group, subjects who lately developed CAA or received IVIG to treat reactivating fever during 10 or more days of illness, were identified as indolent cases (4).

This retrospective study was approved by the Institutional Review Board of the Asan Medical Center (2020-1919). The requirement for informed patient consent was waived.

### Data collection

Demographic and clinical characteristics and laboratory findings were obtained through a review of the medical records. The earliest laboratory test results before the IVIG administration decision were gathered as data.

The measured diameter of coronary arteries during three phases of illness—the acute phase, the subacute phase (from the subsidence of fever to 1 month after illness), and the convalescent phases (between 1 and 3 months after illness), were surveyed. During the acute phase, the results of echocardiography performed before discharge in the defervesced group and the results of echocardiography performed before IVIG administration in the treated group were adopted as data. The Z-score calculation of coronary arteries was based on the study of Olivieri et al. (8). CAA was defined as Z-score ≥2.5 of at least one of the three major coronary arteries—the left main coronary artery, the left anterior descending coronary artery, and the right coronary artery, according to the guidelines of the American Heart Association (1).

### Statistical analysis

Data were presented as mean ± standard deviation or frequency (%). Comparisons of the two groups were performed with Pearson’s Chi-square test for categorical variables and the Student’s *t*-test for continuous variables, including the status of coronary artery diameter. To develop a scoring system for the prediction of spontaneous defervescence, the data sets were randomly divided into two groups, a training or validation set, in a ratio of 2:1. Univariate/multivariate logistic regression analyses were performed with candidate predictors such as demographic, clinical characteristics, and laboratory findings in the training set. The estimated scoring system was evaluated in the validation set. To evaluate the calibration performance of the fitted model, a calibration plot and Hosmer–Lemeshow test were performed on the training set and the validation set. To assess discrimination performance, the area under the curve (AUC) of the receiver operating characteristic (ROC) curve was examined in both data sets. Statistical analyses were conducted using SAS version 9.4 (Cary, NC, United States). A *P* value < 0.05 was regarded as statistically significant.

## Results

Among the 1,441 patients admitted for the management of acute Kawasaki disease during the study period, 1,371 patients were enrolled in the study. Forty-six patients admitted after 10 days of illness and 24 patients whose follow-up ended before the convalescent phase were excluded.

### Demographic and clinical characteristics

Ninety-four of the 1,371 patients (6.9%) experienced spontaneous defervescence after diagnosis of Kawasaki disease, and were classified in the defervesced Kawasaki disease group.

The frequency of complete presentation (75.8 vs. 87.4%, *P* = 0.003) and the presence of rash (72.6 vs. 86.6%, *P* < 0.001) were lower in the defervesced group than in the treated group (Table 1). White blood cell count (*P* < 0.001), neutrophil percentage (*P* < 0.001), and serum level of aspartate aminotransferase (*P* = 0.004), alanine aminotransferase (*P* < 0.001), total bilirubin level (*P* = 0.001), and C-reactive protein (*P* < 0.001), were lower in the defervesced group. Serum albumin level (*P* < 0.001) and sodium level (*P* < 0.001) were higher in the defervesced group. Pyuria was less frequent in the defervesced group (*P* < 0.001).

One patient in the defervesced group was re-admitted for the management of recurrence of fever at home. This 4.8-year-old girl had no CAA after an echocardiographic examination.

### Coronary outcomes

**FIGURE 1:**
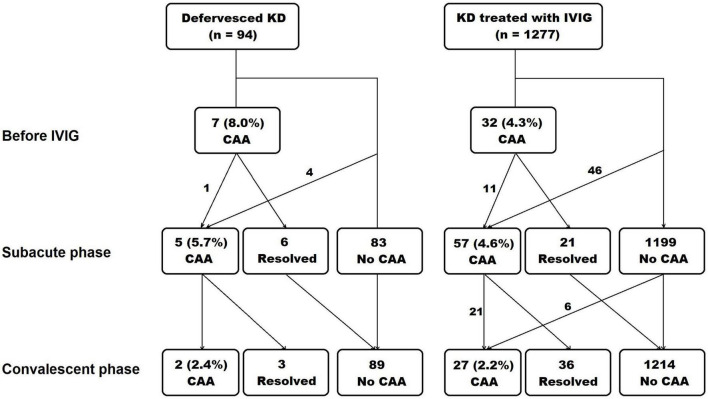
Occurrence of coronary artery aneurysm in each phase of Kawasaki disease. The echocardiography was done at diagnosis and before intravenous immunoglobulin administration, at subacute phase, and at convalescent phase. KD, Kawasaki disease; IVIG, intravenous immunoglobulin; CAA, coronary artery aneurysm.

The occurrence of CAA in each phase is presented in Figure 1. In the defervesced group, 87 subjects underwent echocardiography during admission, and 7 (8.0%) had a CAA. The z score of coronary artery diameters in these seven subjects were all in the small CAA range (≥2.5 to <5.0). The CAA had remained in the subacute phase in one out of the seven patients, but this lesion was resolved in the convalescent phase. In the treated group, 740 subjects (57.9%) underwent echocardiography before the administration of the initial IVIG, and 32(4.3%) had a CAA.

During the subacute and the convalescent phases, the z score of coronary arteries was not different between the defervesced Kawasaki disease and the treated groups (Table 2). The right coronary artery’s measured diameter was smaller in the defervesced group than in the treated group (*P* = 0.047). CAA incidence was 5.7% in the defervesced group and 4.6% in the treated group in the subacute phase (*P* = 0.593) and was 2.5% in the defervesced group and 2.2% in the treated group in the convalescent phase (*P* = 0.924). All coronary artery lesions in the defervesced group were in the range of small CAA (≥2.5 to <5.0). In the treated group, medium or large-sized CAAs were found in 10 subjects during the subacute phase and in six subjects during the convalescent phase.

**TABLE 2:** Coronary artery status during subacute phase and convalescent phase.

	Defervesced KD (*n* = 94)	Treated KD (*n* = 1277)	*P*-value
***During the subacute phase***			
LMCA, mm	2.29 ± 0.42	2.38 ± 0.47	0.089
Z score	0.35 ± 0.90	0.45 ± 0.98	0.357
LAD, mm	1.84 ± 0.44	1.91 ± 0.50	0.252
Z score	0.25 ± 1.11	0.33 ± 1.09	0.495
RCA, mm	1.79 ± 0.45	1.89 ± 0.58	0.107
Z score	–0.27 ± 1.10	–0.10 ± 1.10	0.151
CAA	5 (5.7)	57 (4.6)	0.593
***During the convalescent phase***			
LMCA, mm	2.32 ± 0.42	2.31 ± 0.41	0.832
Z score	0.34 ± 0.89	0.23 ± 0.92	0.260
LAD, mm	1.86 ± 0.35	1.85 ± 0.42	0.866
Z score	0.23 ± 0.91	0.12 ± 0.97	0.311
RCA, mm	1.73 ± 0.37	1.84 ± 0.50	0.047
Z score	–0.50 ± 0.96	–0.28 ± 0.99	0.055
CAA	2 (2.4)	27 (2.2)	0.924

Data are reported as mean ± standard deviation, or number (%).

LMCA, left main coronary artery; LAD, left anterior descending; RCA, right coronary artery; CAA, coronary artery aneurysm.

In the defervesced group, the number of indolent cases was 6 (6.4%), including five subjects with CAA during the subacute phase and one readmission case.

### Scoring model for prediction of spontaneously defervesced Kawasaki disease

All subjects were randomly re-classified into two sets, 914 subjects in the training set and 457 subjects in the validation set. In the training set, logistic regression analyses were performed to predict spontaneous defervescence with variables presented in Table 1. Coronary artery status before initial IVIG was excluded from the analyses due to missing data. The univariate analysis showed that the significant predictors were the presence of a rash [Odds ratio (OR) 0.391, 95% confidence interval (CI) 0.221–0.692, *P* = 0.001], a complete presentation (OR 0.510, 95% CI 0.277–0.940, *P* = 0.031), white blood cell count (OR 0.891, 95% CI 0.836–0.950, *P* < 0.001), neutrophil percentage (OR 0.951, 95% CI 0.937–0.966, *P* < 0.001), serum level of albumin (OR 3.106, 95% CI 1.668–5.783, *P* < 0.001), aspartate aminotransferase (OR 0.989, 95% CI 0.981–0.998, *P* = 0.012), alanine aminotransferase (OR 0.988, 95% CI 0.982–0.995, *P* < 0.001), sodium (OR 1.213, 95% CI 1.098–1.341, *P* < 0.001), and C-reactive protein (OR 0.738, 95% CI 0.668–0.817, *P* < 0.001) (Table 3). The presence of rash (OR 0.509, 95% CI 0.276–0.938, *P* = 0.031), neutrophil percentage (OR 0.975, 95% CI 0.958–0.993, *P* = 0.007), alanine aminotransferase (OR 0.993, 95% CI 0.988–0.999, *P* = 0.026), and C-reactive protein (OR 0.806, 95% CI 0.727–0.893, *P* < 0.001) were also significant predictors in the multivariate analysis.

**TABLE 3:** Predictors of spontaneous defervescence on logistic regression analyses in the training set of 914 subjects.

	Univariate			Multivariate		
	OR	95% CI	*P*-value	OR	95% CI	*P*-value
Rash	0.391	0.221–0.692	0.001	0.509	0.276–0.938	0.031
Complete presentation	0.510	0.277–0.940	0.031			
WBC, × 10^3^/μL	0.891	0.836–0.950	<0.001			
Neutrophil,%	0.951	0.937–0.966	<0.001	0.975	0.958–0.993	0.007
Albumin (mg/dL)	3.106	1.668–5.783	<0.001			
AST, IU/L	0.989	0.981–0.998	0.012			
ALT, IU/L	0.988	0.982–0.995	<0.001	0.993	0.988–0.999	0.026
Na^+^, mmol/L	1.213	1.098–1.341	<0.001			
C-reactive protein (mg/dL)	0.738	0.668–0.817	<0.001	0.806	0.727–0.893	<0.001

OR, Odds ratio; CI, confidence interval; WBC, white blood cell; AST, aspartate aminotransferase; ALT, alanine aminotransferase.

**FIGURE 2:**
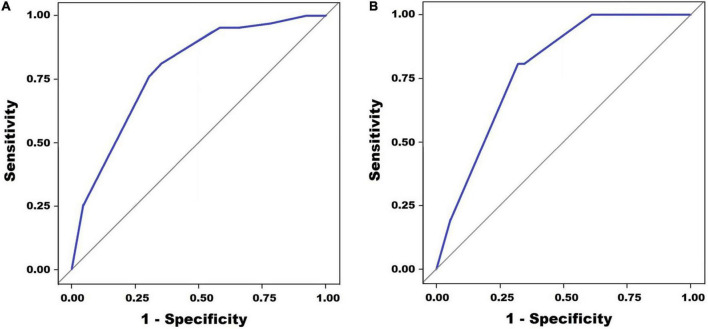
Receiver operating characteristic (ROC) curve analysis for the prediction of spontaneous defervescence. Panel **(A)** shows the multivariate regression model in the training set (AUC = 0.784, 0.737–0.834) and panel **(B)** shows multivariate regression model in the validation set (AUC = 0.787, 0.730–0.843).

A scoring model was developed with categorized variables to increase its usefulness in clinical practice. After ROC curve analyses of neutrophil percentage and C-reactive protein level, the cut-off level for categorization was determined, referring to the Youden index (statistical results not presented). However, in the case of alanine aminotransferase levels, the cut-off level was determined by referring to the Egami scoring system (9) because of the low specificity of the cut-off level (48.1%) by the Youden index. After performing a multivariate logistic regression analysis with newly categorized variables, a score point for variables was determined based on the regression coefficient. The developed scoring model is presented in Table 4. The cut-off level and score points for each constitutional variable were as follows: no rash, 1 point; neutrophil ≤ 65%, 1.5 points; serum alanine aminotransferase level ≤ 80 IU/L, 1.5 points; and serum C-reactive protein level ≤ 10mg/dL, 3 points. The area under the ROC curve was 0.784 (95% CI: 0.737–0.834) (Figure 2). The sum of score points was 7. Under a cut-off level ≥ 5.5 that was determined based on the Youden index, the sensitivity was 77.8%, the specificity was 67.1%, the positive predictive value was 14.9%, and the negative predictive value was 97.6%.

**TABLE 4:** Scoring model of spontaneous defervescence based on the multivariate logistic regression analysis.

Parameter	Cutoff	OR	95% CI	*P*-value	Regression coefficients	Score
No rash		2.084	1.147–3.788	0.016	0.367	1
Neutrophil,%	≤65	3.053	1.625–5.738	0.001	0.558	1.5
ALT, IU/L	≤80	3.342	1.292–8.648	0.013	0.603	1.5
C-reactive protein (mg/dL)	≤10	8.616	2.059–36.058	0.003	1.077	3

OR, Odds ratio; CI, confidence interval; ALT, alanine aminotransferase.

In the validation set, the area under the ROC curve was 0.787 (95% CI: 0.730–0.843) (Figure 2). The sensitivity was 80.7%, the specificity was 68.8%, the positive predictive value was 15.8%, and the negative predictive value was 97.8%.

**FIGURE 3:**
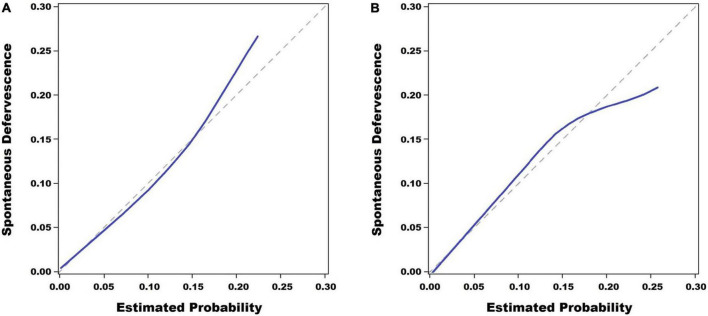
Calibration plots. The scoring system for the prediction of spontaneous defervescence showed a good fitting degree in the Hosmer–Lemeshow test in the training set (*P* = 0.559) **(A)** and it also showed a good fitting degree in the validation set (*P* = 0.704) **(B)**.

The Hosmer–Lemeshow test showed that the estimated and actual probability was highly consistent (training set, *P* = 0.559; validation set, *P* = 0.704) (Figure 3).

## Discussion

This study showed that there was no difference in the occurrence of CAA between patients with spontaneously defervesced Kawasaki disease and patients treated with IVIG. In addition, a scoring model which predicts spontaneous defervescence under the combination of serum C-reactive protein level ≤10 mg/dL and ≥2 conditions of no rash, neutrophil ≤65%, and/or serum alanine aminotransferase level ≤ 80 IU/L, was developed. Although the positive predictive value of this scoring model is not high, it could be used to wait for spontaneous defervescence in the management of acute Kawasaki disease. Currently, the administration of IVIG is the most effective initial treatment and has been conducted safely in most cases of acute Kawasaki disease. However, there is a theoretical risk of blood-borne infection (10) and several adverse effects such as hemolytic anemia (11) and aseptic meningitis (12) have been reported on the administration of IVIG. In addition, the need to change the schedule of live attenuated vaccines after the administration of IVIG (1, 3) is a considerable inconvenience.

The definition of spontaneous defervescence in this study was a body temperature lower than 37.5°C for more than 48 h after arriving at the hospital. This definition was based on our clinical experiences. A body temperature of >37.5°C was suggested as a criterion for persistent fever in Japanese guideline (2). The criterion of 48 h would be an appropriate period, in reference to the recommendations of re-treatment for persistent fever 24–36 h after IVIG administration (1, 2). Additional future investigations and discussion on them will be necessary for the definition of spontaneous defervescence to reach consensus.

In this study, the laboratory characteristics in defervesced Kawasaki disease—lower white blood cell count, neutrophil percentage, level of transaminases, total bilirubin level, C-reactive protein level, and frequency of pyuria and higher hemoglobin concentration, albumin level, and sodium level, implicated a low degree of inflammation. Most of these laboratory characteristics were contrary to those of unresponsiveness to initial IVIG in previous reports (9, 13–16).

In previous studies, an incomplete presentation was reported in 59.2–71.4% of patients with spontaneous defervescence (4, 5). In this study, the incidence of the incomplete presentation was 24.2% which is not significantly different from the approximated incidence of the incomplete presentation in acute Kawasaki disease of 20% (17). Among the principal clinical features, only the frequency of rash was significantly different between the groups, and the absence of rash was a significant variable of spontaneous defervescence in the final prediction model. Notably, the period of investigation does not overlap with the period of the COVID19 pandemic which started in January 2020.

In the report by Hu et al., the incidence of CAA in patients with defervesced Kawasaki disease in the fourth week after onset was 18.9% which was significantly higher than 5.1% in the treated cases (5). There were differences between their study and our study. First, the z score calculation method was different between both studies. Second, cases that received more than one dose of IVIG were excluded from the treated group in their study; it is well-known that the incidence of CAA is higher in retreated patients than the other patients (18).

In the report by Takahashi et al. (4), there were 8 (11.2%) indolent cases among 71 patients with defervesced Kawasaki disease. Among them, 7 that received IVIG between 10 and 29 days after onset had shown either recurrence of fever or re-elevation of C-reactive protein before IVIG administration. However, in this study, only one patient among six (6.4%) indolent cases received IVIG due to the reactivation of fever. Although not statistically significant, the frequency of reactivation of fever after transient subsidence of fever was different between the two studies. Whether or not medium- or higher-dose aspirin (≥30 mg/kg/day) is used during the acute phase might be an explanatory factor. Takahashi et al. reported that oral aspirin (30 mg/kg/day) was administered to all the enrolled patients during the acute stage (4), according to the management guidelines (2). At the Asan Medical Center, medium- or higher-dose aspirin has not been administered in patients with acute Kawasaki disease since 2006, due to the lack of evidence that it has a benefit on the outcome. Recent studies with large study samples have reported that the administration of medium- or higher-dose aspirin does not help prevent coronary artery lesions compared to low-dose aspirin (19, 20). We recommend that the administration of medium- or higher-dose aspirin should not be used when waiting for spontaneous defervescence in acute Kawasaki disease as it may mask the persistent inflammation of the illness.

We do not recommend that the administration of IVIG should be deferred in all patients with acute Kawasaki disease. If fever is identified by a clinical practitioner, IVIG administration must be done immediately. Waiting for spontaneous defervescence should be done under intensive observation by medical professionals while hospitalized at a medical institution.

In this study, seven patients with defervesced Kawasaki disease had a small CAA but the administration of IVIG was not performed as the lesion was expected to improve after systemic inflammation resolution. Fortunately, their CAAs were resolved until the convalescent phase. However, we believe that an additional well-designed study is necessary to determine whether the use of IVIG would help prevent aggravation of CAA in patients with defervesced Kawasaki disease and CAA. In addition, the effect of no administration of IVIG on possible impairment in myocardial deformation during the long-term observation (21) should be investigated in future studies.

This retrospective single institutional study has several limitations. First, the evaluation of the virus infection in incomplete presentation which is more frequent in patients with spontaneous defervescence was not performed according to a designed consistent plan, therefore it could not be included as data in this study. This is a limitation of the retrospective study design. Second, an analysis of the additional benefits of the administration of IVIG to patients with defervesced Kawasaki disease was not possible. Third, the coronary artery status before the decision to administer IVIG could not be used as an independent variable in the logistic regression analyses due to many missing data. Finally, the observation period for the coronary artery outcome was limited to 3 months after the onset of the disease. There have been several reports of Kawasaki disease in the acute phase and developed coronary artery complications in young adults (22–24). However, we think that the subjects in this study are different from the patients with missed diagnosis of Kawasaki disease in that the febrile status of all subjects was observed in the acute phase of illness. Reports of long-term observations in patients not receiving IVIG under the judgment of spontaneous defervescence are very limited. Recently, Dusad et al. reported that none of the 19 patients with defervesced Kawasaki disease showed any coronary abnormalities 3.8 years after the disease onset (25). However, future long-term observational studies with a larger number of patients would be necessary before substantial conclusions can be drawn.

In conclusion, the incidence of CAA in patients with defervesced Kawasaki disease was not different from the treated patients. Using the predictive model developed in this study, patients may wait for spontaneous defervescence after the diagnosis of acute Kawasaki disease under intensive observation by medical staff.

## Data availability statement

The raw data supporting the conclusions of this article will be made available by the authors, without undue reservation.

## Ethics statement

The studies involving human participants were reviewed and approved by Institutional Review Board of the Asan Medical Center (2020-1919). Written informed consent for participation was not provided by the participants’ legal guardians/next of kin because: Personally identifiable information is not included as research data in this retrospective crossectional study. Researchers did not even need to contact patients of their guardians to proceed with the study. The requirement for informed patient consent was waived.

## Author contributions

JY: conceptualization and methodology. HS, HK, MK, and SC: validation. ES and JY: formal analysis. JY, HS, MK, and SC: investigation. ES: original draft. JY, JK, and JB: review and editing. All authors contributed to the article and approved the submitted version.

## References

[1] McCrindle BW Rowley AH Newburger JW Burns JC Bolger AF Gewitz M Diagnosis, treatment, and long-term management of kawasaki disease: a scientific statement for health professionals from the american heart association. *Circulation.* (2017) 135:e927–99. 10.1161/CIR.0000000000000484 28356445

[2] Research Committee of the Japanese Society of Pediatric Cardiology and Cardiac Surgery Committee for Development of Guidelines for Medical Treatment of Acute Kawasaki Disease. Guidelines for medical treatment of acute kawasaki disease: report of the research committee of the japanese society of pediatric cardiology and cardiac surgery (2012 revised version). *Pediatr Int.* (2014) 56:135–58. 10.1111/ped.12317 24730626

[3] Marchesi A De Jacobis IT Rigante D Rimini A Malorni W Corsello G Kawasaki disease: guidelines of the Italian Society of Pediatrics, part I - definition, epidemiology, etiopathogenesis, clinical expression and management of the acute phase. *Ital J Pediatr.* (2018) 44:102. 10.1186/s13052-018-0536-3 30157897 PMC6116535

[4] Takahashi T Sakakibara H Morikawa Y Miura M. Development of coronary artery lesions in indolent Kawasaki disease following initial spontaneous defervescence: a retrospective cohort study. *Pediatr Rheumatol Online J.* (2015) 13:44. 10.1186/s12969-015-0042-8 26530040 PMC4632407

[5] Hu YC Liu HM Lin MT Chen CA Chiu SN Lu CW Outcomes of kawasaki disease children with spontaneous defervescence within 10 days. *Front Pediatr.* (2019) 7:158. 10.3389/fped.2019.00158 31069204 PMC6491630

[6] Hayakawa I Miura M. Giant coronary aneurysms in incomplete kawasaki disease with early spontaneous defervescence. *J Clin Rheumatol.* (2016) 22:40. 10.1097/RHU.0000000000000337 26693626

[7] Newburger JW Takahashi M Gerber MA Gewitz MH Tani LY Burns JC Diagnosis, treatment, and long-term management of kawasaki disease: a statement for health professionals from the committee on rheumatic fever, endocarditis and kawasaki disease, council on cardiovascular disease in the young, american heart association. *Circulation.* (2004) 110:2747–71. 10.1161/01.CIR.0000145143.19711.78 15505111

[8] Olivieri L Arling B Friberg M Sable C. Coronary artery Z score regression equations and calculators derived from a large heterogeneous population of children undergoing echocardiography. *J Am Soc Echocardiogr.* (2009) 22:159–64. 10.1016/j.echo.2008.11.003 19084373

[9] Egami K Muta H Ishii M Suda K Sugahara Y Iemura M Prediction of resistance to intravenous immunoglobulin treatment in patients with Kawasaki disease. *J Pediatr.* (2006) 149:237–40. 10.1016/j.jpeds.2006.03.050 16887442

[10] Cdc. Outbreak of hepatitis C associated with intravenous immunoglobulin administration–United States, October 1993-June 1994. *MMWR Morb Mortal Wkly Rep.* (1994) 43:505–9. 8022396

[11] Luban NL Wong EC Henrich Lobo R Pary P Duke S. Intravenous immunoglobulin-related hemolysis in patients treated for Kawasaki disease. *Tranfusion.* (2015) 55(suppl 2):S90–4. 10.1111/trf.13089 26174904

[12] Kemmotsu Y Nakayama T Matsuura H Saji T. Clinical characteristics of aseptic meningitis induced by intravenous immunoglobulin in patients with Kawasaki disease. *Pediatr Rheumatol Online J.* (2011) 9:28. 10.1186/1546-0096-9-28 21917158 PMC3189389

[13] Kobayashi T Inoue Y Takeuchi K Okada Y Tamura K Tomomasa T Prediction of intravenous immunoglobulin unresponsiveness in patients with Kawasaki disease. *Circulation.* (2006) 113:2606–12. 10.1161/CIRCULATIONAHA.105.592865 16735679

[14] Sano T Kurotobi S Matsuzaki K Yamamoto T Maki I Miki K Prediction of nonresponsiveness to standard high-dose gamma-globulin therapy in patients with acute Kawasaki disease before starting initial treatment. *Eur J Pediatr.* (2007) 166:131–7. 10.1007/s00431-006-0223-z 16896641

[15] Tremoulet AH Best BM Song W Wang S Corinaldesi E Eichenfield JR Resistance to intravenous immunoglobulin in children with Kawasaki disease. *J Pediatr.* (2008) 153:117–21. 10.1016/j.jpeds.2007.12.021 18571548 PMC2526555

[16] Sleeper LA Minich LL McCrindle BM Li JS Mason W Colan SD Evaluation of Kawasaki disease risk-scoring systems for intravenous immunoglobulin resistance. *J Pediatr.* (2011) 158:831–5. 10.1016/j.jpeds.2010.10.031 21168857 PMC3075321

[17] Fukazawa R Kobayashhi J Ayusawa M Hamada H Miura M Mitani Y JCS/JSCS 2020 Guideline on Diagnosis and Management of Cardiovascular Sequelae in Kawasaki Disease. *Circ J.* (2020) 84:1348–407. 10.1253/circj.CJ-19-1094 32641591

[18] Burns JC Capparelli EV Brown JA Newburger JW Glode MP. Intravenous gamma-globulin treatment and retreatment in Kawasaki disease. US/Canadian Kawasaki syndrome stuidy group. *Pediatr Infect Dis J.* (1998) 17:1144–8. 10.1097/00006454-199812000-00009 9877364

[19] Kim GB Yu JJ Yoon KL Jeong SI Song YH Han JW Medium- or higher-dose acetylsalicylic acid for acute Kawasaki disease and patient outcomes. *J Pediatr.* (2017) 184:125–9. 10.1016/j.jpeds.2016.12.019 28043685

[20] Dallaire F Fortier-Morissette Z Blais S Dhanrajani A Basodan D Renaud C Aspirin dose and prevention of coronary abnormalities in Kawasaki disease. *Pediatrics.* (2017) 139:e20170098. 10.1542/peds.2017-0098 28562282

[21] Dedeoglu R Barut K Oztunc F Atik S Adrovic A Sahin S Evaluation of myocardial deformation in patients with Kawasaki disease using speckle-tracking echocardiography during mid-term follow-up. *Cadiol Young.* (2017) 27:1377–85. 10.1017/S1047951117000580 28376935

[22] Kato H Inoue O Kawasaki T Fujiwara H Watanabe T Toshima H. Adult coronary artery disease probably due to childhood Kawasaki disease. *Lancet.* (1992) 340:1127–9. 10.1016/0140-6736(92)93152-d 1359212

[23] Burns JC Shike H Gordon JB Malhotra A Schoenwetter M Kawasaki T. Sequelae of Kawasaki disease in adolescents and young adults. *J Am Coll Cardiol.* (1996) 28:253–7. 10.1016/0735-1097(96)00099-x 8752822

[24] Rizk SRY Said GE Daniels LB Burns JC El Said H Sorour KA Acute myocardial ischemia in adults secondary to missed Kawasaki disease in childhood. *Am J Cardiol.* (2015) 115:423–7. 10.1016/j.amjcard.2014.11.024 25555655 PMC4697961

[25] Dusad S Singhal M Pilania RK Suri D Singh S. CT coronary angiography studies after a mean follow-up of 3.8 years in children with Kawasaki disease and spontaneous defervescence. *Front Pediatr.* (2020) 8:274. 10.3389/fped.2020.00274 PMC7270275 32548085

